# Cancer-related cryptogenic stroke involving the bilateral anterior and the posterior circulations: Diagnostic value of clinical and imaging characteristics

**DOI:** 10.3389/fneur.2022.1032984

**Published:** 2022-12-12

**Authors:** Yifan Xu, Zhuang Wu, Hang Xu

**Affiliations:** ^1^Department of Radiology, Affiliated Hospital 4 of Nantong University, Yancheng, China; ^2^Department of Radiology, Yancheng First People's Hospital, Yancheng, China; ^3^Neurotoxin Research Center of Key Laboratory of Spine and Spinal Cord Injury Repair and Regeneration of Ministry of Education, Department of Neurology, Tongji Hospital, School of Medicine, Tongji University, Shanghai, China; ^4^Department of Neurology, Jiangsu University Affiliated People's Hospital, Zhenjiang, China; ^5^Department of Neurology, Zhenjiang First People's Hospital, Zhenjiang, China

**Keywords:** cancer-related stroke, brain circulations, cryptogenic stroke, DWI, cancer

## Abstract

**Objectives:**

This study analyzed the clinical and imaging characteristics of patients with cancer-related cryptogenic stroke (CCS) involving the bilateral anterior and the posterior circulations (three circulations) and evaluate the diagnostic value of clinical and imaging features for patients with cryptogenic stroke involving three circulations (CST).

**Methods:**

Of the 12,580 patients with acute ischemic stroke, 148 patients with CST from May 2017 to November 2021 were divided into the cancer group (*n* = 81) and the non-cancer group (*n* = 67). Cardiovascular risk factors, diffusion-weighted imaging patterns of cryptogenic stroke, blood routine, coagulation routine, and biochemical routine were compared between the two groups. Multivariable logistic regression and receiver operating characteristic (ROC) curve analysis were used to determine associations between the two groups.

**Results:**

Compared with the non-cancer group, the cancer group exhibited higher D-dimer levels (*P* < 0.001), fibrin degradation product (FDP, *P* < 0.001), international normalized ratio (INR, *P* = 0.014), neutrophil to lymphocyte ratio (NLR, *P* < 0.001), platelets to lymphocyte ratio (PLR, *P* = 0.001), activated partial thromboplastin time (APTT, *P* = 0.039), more frequent multiple lesions in three circulations (*P* < 0.001) and lower lymphocytes (*P* < 0.001), red blood cells (*P* < 0.001), and thrombin time (TT, *P* = 0.034). Furthermore, D-dimer [area under the curve (AUC) = 0.915, *P* < 0.001)], FDP (AUC = 0.923, *P* < 0.001), INR (AUC = 0.617, *P* = 0.014), NLR (AUC = 0.700, *P* < 0.001), PLR (AUC = 0.658, *P* = 0.001), and multiple lesions in three circulations (AUC = 0.786, *P* < 0.001) had potential diagnostic value in cryptogenic stroke. When combining these 6 parameters, the predictive power was improved (AUC = 0.949, *P* < 0.001).

**Conclusion:**

Cryptogenic stroke involving three circulations with cancer has unique clinical features, and these potential diagnostic indicators could help patients identify CCS earlier.

## Introduction

Cancer and stroke are the leading causes of death and disability worldwide (1–3). Stroke is the second most common central nervous system complication among cancer patients. A previous study indicated that ~15% of patients with cancer had a stroke at the time of death which was significantly higher than the incidence of stroke in the general population (7.6%) (4). The stroke rate in patients with cancer was twice as high as in the general population (5–7). The incidence of stroke in patients with cancer was also increasing due to the prolonged survival of cancer patients (8). Cancer-related stroke could influence these patients' quality of life seriously.

Previous studies indicated that cancer patients without traditional mechanisms of stroke after thorough examinations were considered to be patients with cancer-related cryptogenic stroke (CCS) (9, 10). Unlike traditional stroke, the occurrence of CCS is associated with hypercoagulable states, non-bacterial thrombotic endocarditis, cancer therapy, etc. (11). A previous study indicated that patients with CCS exhibited high plasma D-dimer levels and multi-vascular ischemic stroke lesions (12). A prospective study demonstrated that these two features might be predictors of occult cancer (13). In addition, patients with cryptogenic stroke had a worse prognosis than those with conventional stroke (14, 15). Previously, ~75% of the patients with three-territory signs (patients with stroke lesions involving the bilateral anterior and the posterior circulations) had a cancer-related stroke. However, the results are waiting to be confirmed due to its small sample size (16). In addition, the clinical and imaging features of CCS involving three circulations (CCST) have not been well studied.

Accordingly, this study aimed (1) to compare the clinical and imaging characteristics of patients with and without CCST and (2) to evaluate the diagnostic value of clinical and imaging characteristics for patients with cryptogenic stroke involving three circulations (CST).

## Methods

### Patients and stroke etiology

This retrospective study enrolled 12,580 patients with acute ischemic stroke from the Fourth Affiliated Hospital of Nantong University between May 2017 and November 2021. Cryptogenic stroke was defined as having no specific attributable cause after a comprehensive evaluation of common causes (17). Cancer patients with unconventional stroke mechanisms could be considered patients with CCS (9). Active cancer was defined as newly diagnosed, metastatic, progressive, or recurrent within 6 months prior to this enrollment. In addition, patients with active cancer, squamous skin cell carcinoma, or patients with localized basal cell carcinoma need to be excluded (18, 19). Inclusion criteria of cancer patients with CST were as follows: (1) age > 18 years; (2) diagnosed as acute ischemic stroke (within 72 h of onset symptoms) involving three circulations; (3) classified as cryptogenic stroke; (4) with active cancer; and (5) with complete clinical information. Exclusion criteria of cancer patients with CST were as follows: (1) recently with myocardial infarction, anticoagulant therapy, chronic kidney disease, or other diseases that might affect the coagulation index; (2) with inactive cancer, a primary brain tumor, or an undiagnosed tumor; and (3) with incomplete medical records. Inclusion criteria of non-cancer patients with CST were as follows: (1) age > 18 years; (2) diagnosed as acute ischemic stroke (within 72 h of onset symptoms) involving three circulations; (3) classified as cryptogenic stroke; (4) without cancer; and (5) with complete clinical information. Exclusion criteria of non-cancer patients with CST were as follows: (1) patients with myocardial infarction, anticoagulant therapy, chronic kidney disease, or other diseases that might affect the coagulation index; (2) with a primary brain tumor; and (3) with incomplete medical records. This study was approved by the Ethical Committee of the institution [(2021) - (k-104)], and all informed consents were obtained.

### Ischemic stroke pattern

The cerebral vascular (including a total of 23 vascular territories) distribution is divided into the bilateral anterior and the posterior circulations (20, 21). Based on observed diffusion-weighted imaging (DWI) patterns, ischemic stroke lesions were classified into (1) a single lesion in a single one vascular territory, (2) scattered lesions in one vascular territory, (3) multiple lesions in multiple vascular territories, and (4) multiple lesions in three circulations (it is defined as all three circulations with multiple lesions). All imaging assessments are carried out independently by two experienced physicians.

### Clinical information

This study collected data on patients during their hospitalization, including baseline characteristics (including age, gender, hypertension, diabetes, hyperlipidemia, and smoking), medical history (including recurrence, metastasis, and treatment of cancer patients), and medical examination (including echocardiography, ECG, extracranial vascular examination, and pathological reports of cancer). In addition, this study collected complete blood count [including white blood cell count, red blood cells, neutrophils, lymphocytes, platelet count, serum lactate dehydrogenase (LDH)], coagulation routine during admission without anticoagulant treatment [including D-dimer, international normalized ratio (INR), fibrinogen (FIB), fibrin degradation product (FDP), thrombin time (TT), activated partial thromboplastin time (APTT), antithrombin-III (AT-III)], and biochemical routine (including total protein, globulin, albumin, triglyceride, and total cholesterol levels).

### Statistical analysis

Quantitative data were expressed as mean ± standard deviation or median (interquartile range) as appropriate. All categorical data were presented as a percentage. Values were compared using the Mann–Whitney *U* test or the independent *t*-test for quantitative variables as appropriate. Categorical variables were analyzed for the chi-square test or Fisher's exact test as appropriate. The independent non-collinear clinical features that were significantly correlated with CST at the univariate level (*P* < 0.01) were then included in the multivariate logistic regression model. Receiver operating characteristic (ROC) curve analysis and Youden's index were performed to determine the best cutoff value for some factors that differentiated cryptogenic stroke with or without cancer. SPSS software version 26 (IBM Corp, Armonk, NY, United States) was used for data analyses. Statistical significance was considered to be *P* < 0.05.

## Results

**Figure 1:**
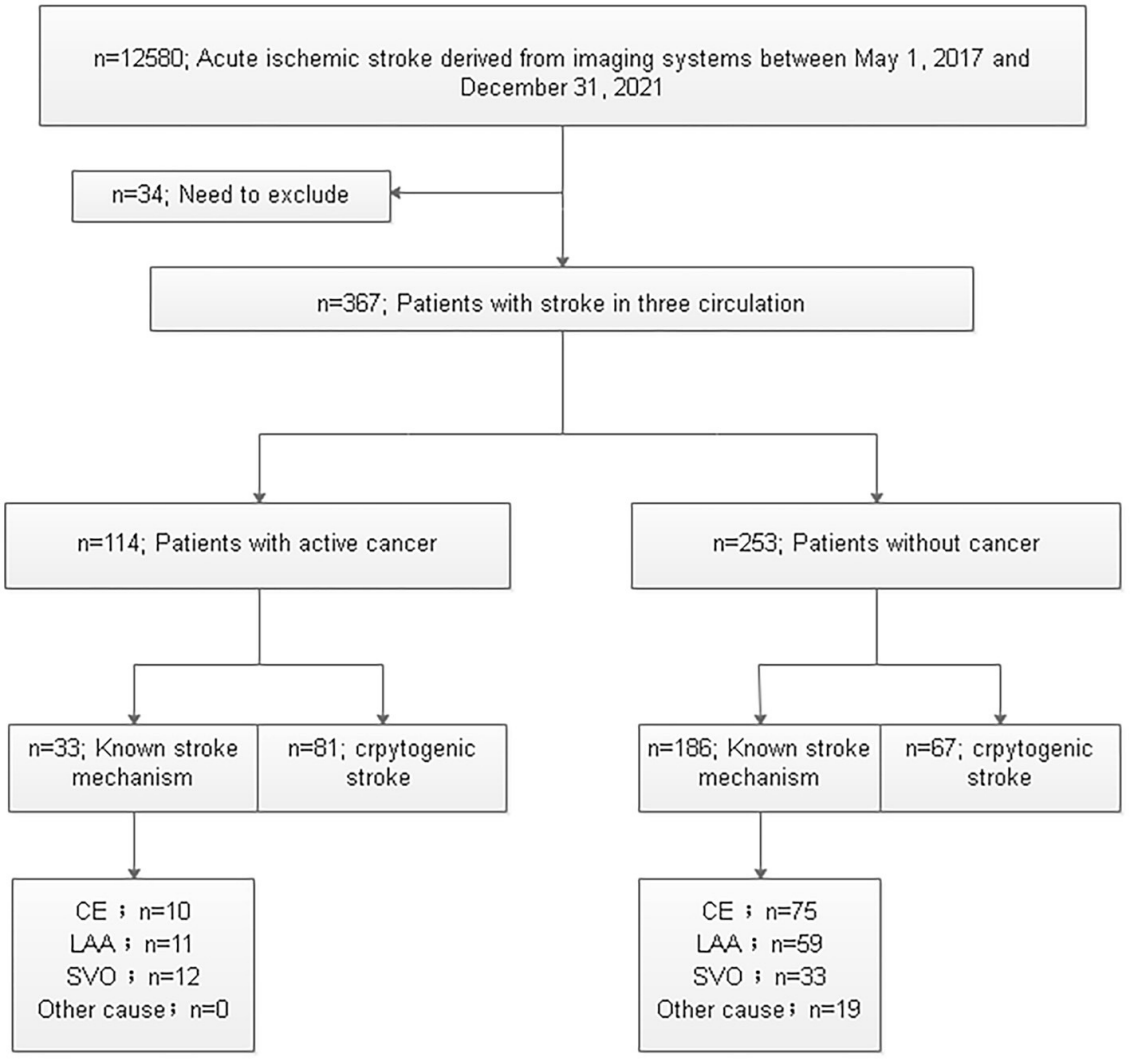
Flowchart of patient selection. CE, cardioembolism; LAA, large-artery atherosclerosis; SVO, small-vessel occlusion.

Of all 12,580 patients diagnosed with ischemic stroke on MRI, 2.92% of patients (367/12,580) had ischemic stroke involving three circulations. According to the Trial of ORG 10172 in Acute Stroke Treatment (TOAST) criteria (22), 367 patients were classified into the subtypes that incorporated large-artery atherosclerosis, small-vessel occlusion, cardioembolism, stroke of other determined cause, and cryptogenic stroke (Figure 1). Notably, 148 patients with cryptogenic stroke were selected from 367 patients with ischemic stroke involving three circulations. A total of 148 patients with CST were divided into the cancer group (81, 54.37%) and the non-cancer group (67, 45.27%) based on whether they had active cancer or not (Figure 1). The incidence of cryptogenic stroke in all patients with active cancer was 71.05% (81/114), and the incidence of cryptogenic stroke in all patients with non-cancer was 26.48% (67/253) (*P* < 0.001).

The most common cancer in patients with CCST was lung cancer (29/81, 35.80%), followed by pancreatic cancer (9/81, 11.11%), gastroduodenal cancer (9/81, 11.11%), esophageal cancer (8/81, 9.99%), other cancers (5/81, 6.17%), prostate cancer (4/81, 4.94%), cholangiocarcinoma (3/81, 3.70%), breast cancer (3/81, 3.70%), ovarian cancer (3/81, 3.70%), bladder cancer (2/81, 2.47%), gallbladder cancer (2/81, 2.47%), cervical cancer (2/81, 2.47%), colon cancer (1/81, 1.23%), and kidney cancer (1/81, 1.23%). Of note, 45.68% (37/81) of patients had lymph node metastases, and 60.49% (49/81) of patients had distant metastases. Notably, 20.99% (17/81) of patients were treated with surgery, and 28.40% (23/81) of patients were treated with chemotherapy or radiotherapy.

Among the cancer group, 41 (50.61%) were men, and the mean age was 68.84 ± 8.14 years. Among the non-cancer group, 42 (62.69%) were men, and the mean age was 67.48 ± 12.93 years. Baseline characteristics were compared between cancer and non-cancer groups. There were no statistical differences between the two groups in any of the baseline characteristics (Table 1).

**Table 1:** Clinical characteristics of participants.

**Cryptogenic**	**With**	**Without**	***P*-value**
**stroke**	**cancer (81)**	**cancer (67)**	
Age, years	68.84 ± 8.14	67.48 ± 12.93	0.141
Male, %	41 (50.61)	42 (62.69)	0.142
Hypertension, %	43 (53.08)	33 (49.25)	0.642
Hyperlipidemia, %	17 (20.99)	17 (25.37)	0.528
Diabetes mellitus, %	31 (38.27)	36 (53.73)	0.060
Current smoking, %	11 (13.58)	11 (16.42)	0.602

**Figure 2:**
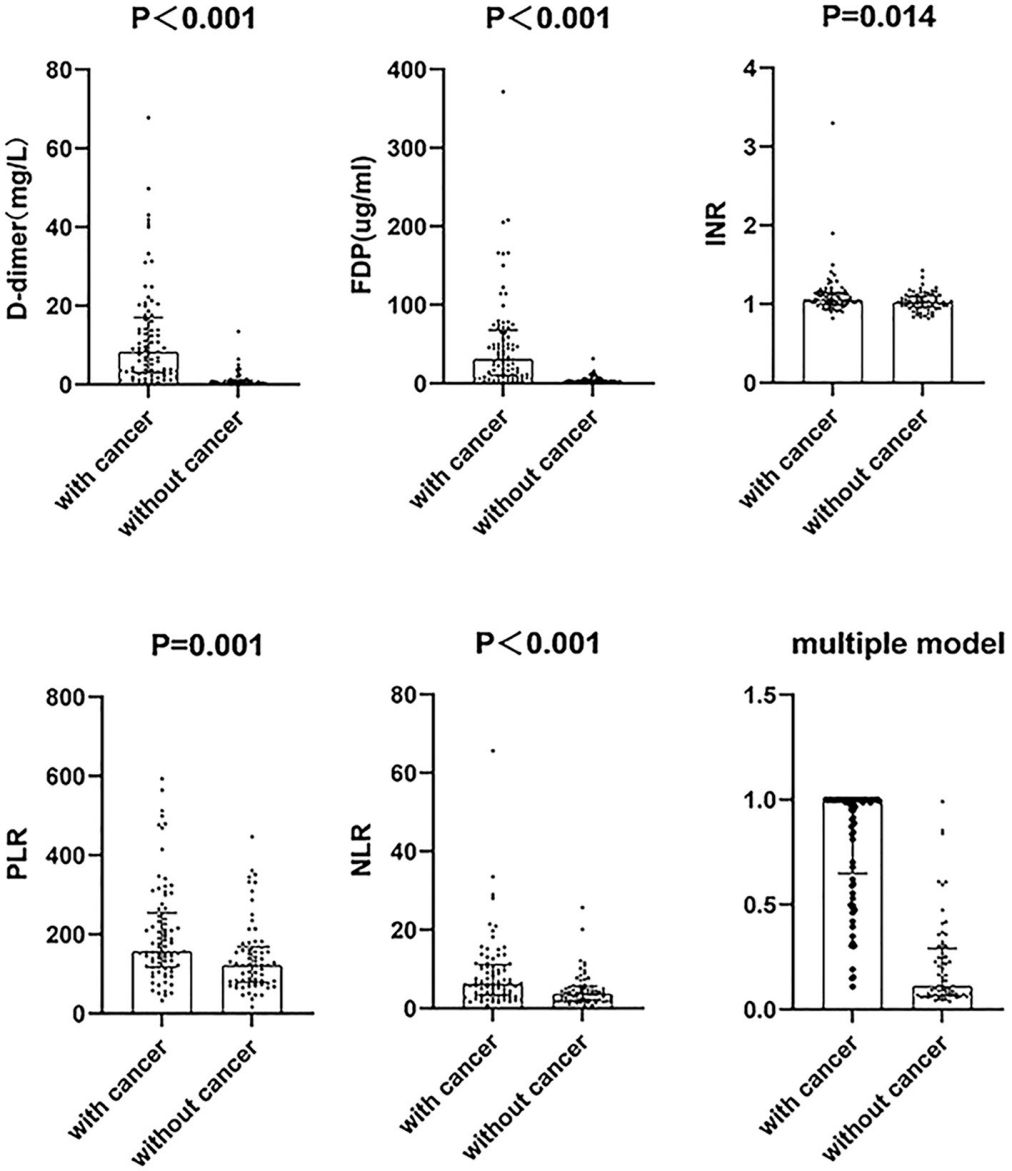
The difference between cancer and non-cancer groups in six parameters of cryptogenic stroke involving three circulations patients. The distribution of the data points including the median with an interquartile range. NLR, neutrophil to lymphocyte ratio; PLR, platelets to lymphocyte ratio; FDP, fibrin degradation product; INR, international normalized ratio.

For clinical characteristics (Table 2), the cancer group exhibited higher neutrophil to lymphocyte ratio (NLR, *P* < 0.001), platelets to lymphocyte ratio (PLR, *P* = 0.001), D-dimer (*P* < 0.001), FDP (*P* < 0.001), INR (*P* = 0.014), and APTT (*P* = 0.039) than the non-cancer group (Figure 2). In addition, the cancer group had lower lymphocytes (*P* < 0.001), red blood cells (*P* < 0.001), and TT (*P* = 0.034) than the non-cancer group.

**Table 2:** Characteristics of patients with cryptogenic stroke with and without cancer.

**Cryptogenic**	**With**	**Without**	***P*-value**
**stroke**	**cancer (81)**	**cancer (67)**	
White blood cells x 10^9^/L	7.38 (6.06, 10.45)	7.15 (5.61, 10.44)	0.376
Lymphocytes x 10^9^/L	0.92 (0.66, 1.22)	1.45 (1.07, 1.97)	**<0.001**
Red blood cells x 10^12^/L	3.64 (3.07, 4.11)	4.25 (3.37, 4.64)	**<0.001**
Neutrophils x 10^9^/L	6.38 (4.07, 8.87)	5.00 (3.28, 8.01)	0.077
Platelets x 10^11^/L	161.10 (117.60, 217.55)	171.00 (138.10, 243.10)	0.261
NLR	6.19 (3.33, 11.13)	3.72 (1.94, 5.55)	**<0.001**
PLR	157.00 (117.06, 254.46)	121.65 (78.05, 167.54)	**0.001**
LDH, U/L	397.95 (279.25, 596.18)	377.00 (204.30, 510.00)	0.190
TPO, g/L	62.74 ± 8.98	64.32 ± 8.38	0.275
Albumin, g/L	34.14 ± 6.23	35.79 ± 6.26	0.111
Globulin, g/L	29.27 ± 5.38	28.41 ± 4.39	0.197
Total cholesterol, mmol/L	4.35 ± 1.50	4.25 ± 1.78	0.720
Triglyceride, mmol/L	1.34 (1.02, 1.98)	1.38 (1.14,1.98)	0.310
D-dimer, mg/L	8.29 (3.02, 17.06)	0.62 (0.31, 1.13)	**<0.001**
FIB, g/L	2.70 (1.65, 3.64)	2.90 (2.50, 3.58)	0.054
FDP, ug/mL	30.80 (10.00, 67.90)	3.10 (2.10, 4.60)	**<0.001**
INR	1.05 (1.00, 1.14)	1.02 (0.96, 1.10)	**0.014**
TT, s	15.90 (14.80, 17.35)	16.70 (15.30, 17.80)	**0.034**
APTT, s	27.60 (25.70, 30.70)	26.30 (23.80, 29.70)	**0.039**
AT-III, %	86.71 ± 19.38	88.29 ± 16.47	0.599

NLR, neutrophil to lymphocyte ratio; PLR, platelets to lymphocyte ratio; LDH, lactic dehydrogenase; TPO, total protein; FIB, fibrinogen; FDP, fibrin degradation product; INR, international normalized ratio; TT, thrombin time; APTT, activated partial thromboplastin time; AT-III, antithrombin-III. Bold signifies significant results.

**Figure 3:**
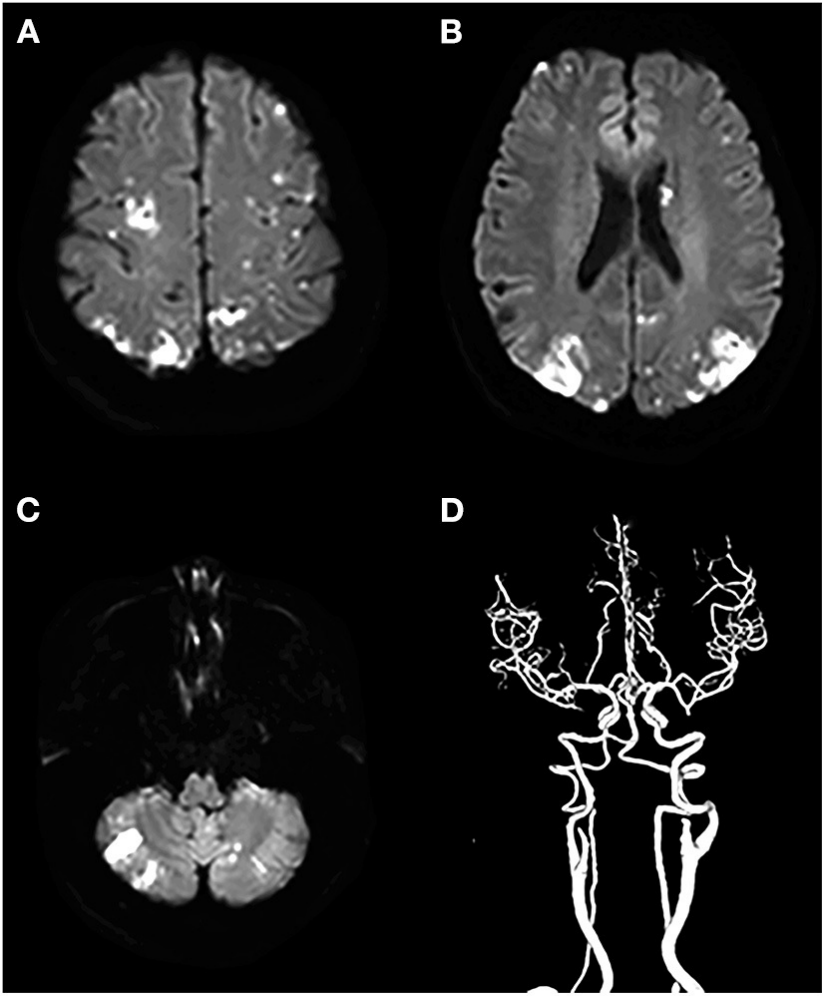
A cryptogenic stroke involving three circulations in a 72-year-old woman with a malignant tumor of the abdomen. The patient had no traditional risk factors for ischemic stroke such as atrial fibrillation, hypertension, diabetes mellitus, etc. **(A–C)** DWI images show multiple acute lesions in multiple vascular territories (in the bilateral anterior and the posterior circulations), and **(D)** shows no meaningful stenosis on CTA.

When evaluating the pattern of cryptogenic stroke lesions on DWI studies, cryptogenic stroke as a single lesion in one vascular territory of left anterior circulations was significantly less often observed in patients with CCST than in patients with CST without cancer. Similar results were also observed in the right anterior and posterior circulations (Table 3). The incidence of multiple lesions in multiple vascular territories in multiple circulations (two or three circulations) (Figure 3) of the cancer group was significantly higher (50/81, 61.71%) than that of the non-cancer group (7/67, 10.54%) (*P* < 0.001). The incidence of multiple lesions in three circulations of the cancer group (62/81, 76.54%) was higher than that of the non-cancer group (13/67, 19.40%) (*P* < 0.001) (Table 3).

**Table 3:** The DWI patterns of patients with cryptogenic stroke with and without cancer.

**Ischemic lesion**	**With**	**Without**	***P*-value**
**pattern (DWI)**	**cancer (81)**	**cancer (67)**	
**Left anterior circulations**			
**A single lesion in one vascular territory**			
<15 mm	4 (4.93%)	29 (43.28%)	**<0.001**
>15 mm	4 (4.93%)	2 (2.99%)	0.856
Scattered lesions in one vascular territory	24 (29.63%)	23 (34.32%)	0.541
Multiple lesions in multiple vascular territories	49 (60.49%)	13 (19.40%)	**<0.001**
**Right anterior circulations**			
**A single lesion in one vascular territory**			
<15 mm	12 (14.81%)	33 (49.25%)	**<0.001**
>15 mm	2 (2.47%)	2 (2.99%)	1.000
Scattered lesions in one vascular territory	31 (38.27%)	27 (40.30%)	0.801
Multiple lesions in multiple vascular territories	36 (44.44%)	5 (7.46%)	**<0.001**
**Posterior circulations**			
**A single lesion in one vascular territory**			
<15 mm	15 (18.52%)	38 (56.72%)	**<0.001**
>15 mm	0 (0.00%)	7 (10.44%)	**0.010**
Scattered lesions in one vascular territory	10 (12.35%)	9 (13.43%)	0.844
**Multiple lesions in multiple vascular territories**			
Two or three circulations	50 (61.73%)	7 (10.45%)	**<0.001**
Two circulations	29 (35.80%)	7 (10.45%)	**<0.001**
Three circulations	21 (25.93%)	0 (0.00%)	**<0.001**
Multiple lesions in three circulations	62 (76.54%)	13 (19.40%)	**<0.001**

DWI, diffusion-weighted imaging. Bold signifies significant results.

A total of 12 potential markers of CCST were selected based on the univariate logistic regression (Table 4). We used the ROC curve to evaluate the value of clinical and imaging parameters in distinguishing cancer and non-cancer groups. We found 11 parameters with potential diagnostic values (Table 4). The optimal cut-off for ROC curves analysis of the D-dimer level was 2.25, with sensitivity and specificity of 82.70 and 86.60%, respectively. The optimal FDP cut-off was 7.75, with sensitivity and specificity of 81.50 and 88.10%, respectively. The optimal INR cut-off was 1.03, with sensitivity and specificity of 63.00 and 58.20%, respectively. The optimal PLR cut-off was 131.75, with sensitivity and specificity of 69.10 and 58.20%, respectively. The optimal NLR cut-off was 5.05, with sensitivity and specificity of 64.20 and 73.10%, respectively. The optimal cut-off for multiple lesions in three circulations is 0.5, with sensitivity and specificity of 76.50 and 80.60%, respectively (Table 4).

**Table 4:** Univariate logistic regression and ROC analysis for CCS involving three circulations.

	**Univariate logistic regression**			**ROC analysis**					
**Risk factors**	**OR**	**95% CI**	***P-*** **value**	**AUC**	**95% CI**	***P*** **-value**	**Cut-off**	**Sensitivity (%)**	**Specificity (%)**
**Blood routine**	
Red blood cells	0.68	0.48–0.97	**0.031**	0.67	0.58–0.76	**<0.001**	2.09	97.50	10.40
Lymphocytes	0.20	0.10–0.40	**<0.001**	0.76	0.68–0.84	**<0.001**	3.21	1.20	100.00
NLR	1.15	1.06–1.25	**0.001**	0.70	0.62–0.78	**<0.001**	5.05	64.20	73.10
PLR	1.01	1.00–1.01	**0.003**	0.66	0.57–0.75	**0.001**	131.75	69.10	58.20
**Coagulation routine**	
D-dimer	1.74	1.38–2.19	**<0.001**	0.92	0.87–0.96	**<0.001**	2.25	82.70	86.60
FIB	0.77	0.59–0.99	**0.045**	0.59	0.50–0.68	0.054	3.59	30.90	76.10
FDP	1.23	1.12–1.35	**<0.001**	0.92	0.88–0.97	**<0.001**	7.75	81.50	88.10
INR	28.24	1.64–487.78	**0.022**	0.62	0.53–0.71	**0.014**	1.03	63.00	58.20
TT	1.01	0.92–1.07	0.722						
APTT	1.06	0.99–1.14	0.106						
**Multiple lesions in multiple vascular territories**			
Two circulations	4.78	1.93–11.82	**0.001**	0.63	0.54–0.72	**0.008**	0.50	35.80	89.60
Three circulations	0.47	0.39–0.57	**<0.001**	0.63	0.54–0.72	**0.007**	0.50	25.90	100.00
Two or three circulations	13.83	5,61–34.07	**<0.001**	0.76	0.68–0.84	**<0.001**	0.50	61.70	89.60
Multiple lesions in three circulations	13.56	6.12–30.00	**<0.001**	0.79	0.71–0.86	**<0.001**	0.50	76.50	80.60

CCS, cancer-related cryptogenic stroke; ROC, receiver operating characteristic; OR, odds ratio; CI, confidence interval; AUC, area under the curve; NLR, neutrophil to lymphocyte ratio; PLR, platelets to lymphocyte ratio; FIB, fibrinogen; FDP, fibrin degradation product; INR, international normalized ratio; TT, thrombin time; APTT, activated partial thromboplastin time. Bold signifies significant results.

**Figure 4:**
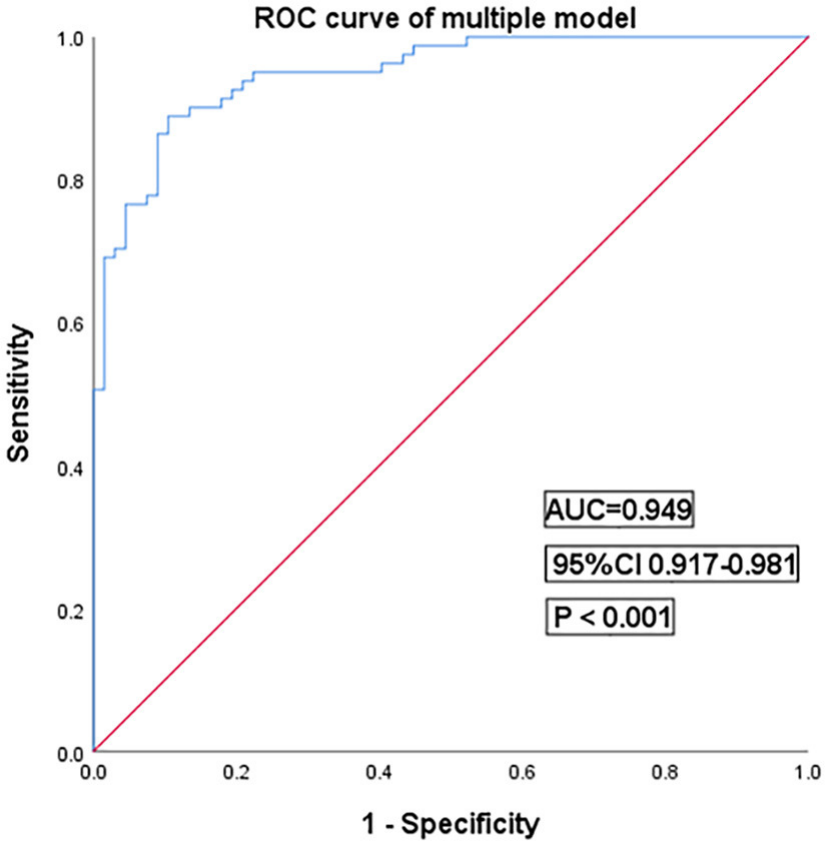
Receiver operating characteristic curves for the multiple models of cryptogenic stroke involving three circulations patients. AUC, area under the curve; CI, confidence interval.

Then, in multivariate logistic regression analysis, variables were statistically significant (*P* < 0.01) at the univariate analysis level as shown in Table 5. Moreover, INR is considered meaningful in clinical practice, and it was also enrolled in multivariate logistic regression analysis. The model had a better diagnostic value with an area under the curve (AUC) of 0.95 (95% CI: 0.92–0.98). At a cut-off value of 0.42, the sensitivity and specificity of the multiple models were 88.9 and 89.5%, respectively (Figure 4).

**Table 5:** Multivariate logistic regression for CCS involving three circulations.

**Risk factors**	**OR**	**95% CI**	***P*-value**
NLR	1.03	0.97–1.10	0.378
PLR	1.01	1.00–1.01	0.069
D-dimer	0.96	0.66–1.41	0.833
FDP	1.23	1.05–1.44	**0.011**
INR	2.16	0.12–38.60	0.602
Multiple lesions in three circulations	3.83	1.33–11.09	**0.013**

CCS, cancer-related cryptogenic stroke; OR, odds ratio; CI, confidence interval; NLR, neutrophil to lymphocyte ratio; PLR, platelets to lymphocyte ratio; FDP, fibrin degradation product; INR, international normalized ratio. Bold signifies significant results.

## Discussion

The main purpose of this study was to analyze the clinical and imaging characteristics of patients with CCST and evaluate the diagnostic value of clinical and imaging features for patients with CST. The DWI patterns of cryptogenic stroke, blood routine, and coagulation routine of cancer patients with CST are significantly different from non-cancer patients with CST, and these parameters can aid in diagnosing patients with CCST.

As yet, there is still no consensus on how to identify cancer risk in patients with acute stroke (23). In terms of the incidence of cancer in ischemic stroke, a study carried out in the United States revealed that approximately 10% of ischemic stroke inpatients had cancer (24). Another research showed that the rate of ischemic stroke patients with cancer was ~12% (25), which was smaller than the results of our research. Our study reported that 31.06% of ischemic strokes involving three circulations patients have comorbid cancer. Additionally, 54.37% of patients with CST have comorbid cancer, which was significantly higher than the incidence of stroke patients with cancer. Previous studies proposed that three territory signs indicated a possible hypercoagulable stroke due to cancer when no other cause is apparent (16). These studies indicated that there was a strong correlation between cancer and CST. Accordingly, we need to pay more attention to patients with CST.

Our study showed that patients with CCST had unique clinical and imaging features with higher D-dimer, FDP, INR, PLR, and NLR and more frequent multiple lesions in three circulations compared to patients with non-cancer CST. The possible reasons may be as follows. First, in the OASIS-Cancer study, they reported that cancer cell-derived extracellular vesicle levels correlated with D-dimer levels, a non-specific marker of hypercoagulability, and that cancer cell-derived extracellular vesicle levels were highest among patients with cryptogenic stroke (26). These mechanisms may lead to elevated D-dimer in patients with CCST. In addition, the D-dimer level is also associated with the severity and prognosis of cancer (13, 14, 27). These could also explain the high proportion of patients with CCST in advanced stages in this study. Second, Kono et al. (28) showed a correlation between increased FDP and cancer-related stroke, which indicated that FDP could be used as a potential biomarker to identify CCST from CST. Third, our study illustrated a higher INR in patients with CCS than in the non-cancer group. Previous studies showed that the INR of the cancer-related stroke group was higher than the INR of the non-cancer group (29, 30), which was similar to our study. Furthermore, the PLR is an easily applied blood test based on platelet and lymphocyte counts. Platelet aggregation and degranulation and the subsequent release of platelet granules and extracellular vesicles are thought to be important determinants of tumor growth. In turn, platelets promote tumor growth through proliferative signaling, anti-apoptotic effects, and angiogenic factors (31). Moreover, the release of immune mediators could cause a significant immunosuppressive effect and lymphocyte counts reduce. These reasons lead to an increased PLR in patients with cancer (32). Studies had reported a correlation between increased PLR and thromboembolism in patients with cancer (33, 34). The above theory could explain why PLR levels were significantly higher in patients with CCST than PLR in CST patients without cancer in our study. At the same time, NLR and PLR are easy to calculate from blood routine, and they are used as new predictors of thrombotic events (33). A retrospective analysis study showed a strong correlation between NLR and CCS (35), which could also explain why NLR was significantly higher in the cancer group than in the non-cancer group. Therefore, we included NLR in this study as an indicator to differentiate CCST from CST. Finally, several studies had shown that cancer-related stroke was associated with stroke in multiple vascular territories (36, 37). However, the above studies did not have a relationship between multi-vascular stroke and cancer. The results of this study showed for the first time that patients with CST had a significantly higher rate of active cancer than non-cancerous patients (76.54 vs. 19.40%). This finding could have a high diagnostic value in clinical practice. Our study expanded previous studies and demonstrated that these risk factors can be used to discriminate patients with CCST from patients with CST.

This study still had some limitations. First, our study was a retrospective analysis with a relatively small sample size, which might lead to selection bias. Second, patients with cryptogenic stroke involving a single circulations were not included in this study. In addition, ischemic stroke patients without MRI scanning due to the severity of their disease were not enrolled in this study. This might result in selection bias. Third, the mechanism of cancer-related hypercoagulable state was not fully understood, and there were large differences between individual cancer patients.

## Conclusion

There was a high incidence of cancer in patients with stroke involving three circulations and a high incidence of cancer in patients with cryptogenic stroke involving three circulations. Blood routine, coagulation routine, and DWI patterns of cryptogenic stroke are potential biomarkers to identify CCST.

## Data availability statement

The original contributions presented in the study are included in the article/Supplementary material, further inquiries can be directed to the corresponding authors.

## Ethics statement

The studies involving human participants were reviewed and approved by Affiliated Hospital 4 of Nantong University. The patients/participants provided their written informed consent to participate in this study. Written informed consent was obtained from the individual(s) for the publication of any potentially identifiable images or data included in this article.

## Author contributions

YX contributed to the design of this study, helped in drafting the manuscript for intellectual content, had a major role in the acquisition of data, and contributed to the analysis and interpretation of the data. ZW and HX contributed to the design of the study and helped in drafting the manuscript for intellectual content. All authors contributed to the article and approved the submitted version.
